# Emerging Mechanisms of Innate Immunity and Their Translational Potential in Inflammatory Bowel Disease

**DOI:** 10.3389/fmed.2018.00032

**Published:** 2018-02-19

**Authors:** Daniele Corridoni, Thomas Chapman, Tim Ambrose, Alison Simmons

**Affiliations:** ^1^MRC Human Immunology Unit, Weatherall Institute of Molecular Medicine, John Radcliffe Hospital, University of Oxford, Oxford, United Kingdom; ^2^Translational Gastroenterology Unit, John Radcliffe Hospital, University of Oxford, Oxford, United Kingdom

**Keywords:** innate immunity, pattern-recognition receptors, toll-like receptors, NOD-like receptors, inflammatory bowel disease

## Abstract

Activation of the innate immune system through pattern-recognition receptor (PRR) signaling plays a pivotal role in the early induction of host defense following exposure to pathogens. Loss of intestinal innate immune regulation leading aberrant immune responses has been implicated in the pathogenesis of inflammatory bowel disease (IBD). The precise role of PRRs in gut inflammation is not well understood, but considering their role as bacterial sensors and their genetic association with IBD, they likely contribute to dysregulated immune responses to the commensal microbiota. The purpose of this review is to evaluate the emerging functions of PRRs including their functional cross-talk, how they respond to mitochondrial damage, induce mitophagy or autophagy, and influence adaptive immune responses by interacting with the antigen presentation machinery. The review also summarizes some of the recent attempts to harness these pathways for therapeutic approaches in intestinal inflammation.

## The Innate Immune System and Pathogen Sensing

The innate immune system represents the first line of defense against pathogens, providing an immediate response to infection, and is conserved throughout evolution (1, 2). In vertebrates, the innate immune system also serves to prime the adaptive immune system, generating long-lasting immunological memory. The critical importance of the innate immune system is underlined by the observation that defects can be lethal to the host (2) or associated with inflammatory disorders, such as inflammatory bowel disease (IBD).

The innate immune system faces two key challenges. First, it is faced with a near constant barrage of microorganisms. Second, it must accurately and rapidly discriminate between non-infectious self and infectious pathogen. To achieve this, it relies upon a relatively small number of pattern-recognition receptors (PRRs) that recognize features common to many pathogens, known as pathogen-associated molecular patterns (2). Pathogen-associated molecular patterns are typically critical for survival of the pathogen and thus cannot be mutated by the pathogen to avoid detection; consequently, they are often invariant across entire classes of pathogens (3).

Pattern-recognition receptors are germline-encoded receptors that are expressed on the cell surface, in intracellular compartments such as the cytosol or endosomes, or secreted into serum or tissue fluid. PRRs are classified according to their function, localization, and ligand specificity. Two main classes of PRRs have been described in mammalian cells, namely membrane bound receptors, including toll-like receptors (TLRs) and C-type lectin receptors (CLRs), and cytoplasmic receptors, including nucleotide-binding oligomerization domain (NOD)-like receptors (NLRs), RIG-I-like receptors (RLRs), AIM2-like receptors, and the more recently identified cyclic GMP-AMP synthase (cGAS). PRRs often act in synergy allowing rapid amplification of the initial immune response. The varied location of PRRs provides the ability to detect pathogens invading a range of cellular compartments. Multiple PRRs are capable of recognizing a single microorganism, which may present a broad array of antigenic ligands, ensuring a robust immune response. PRRs also recognize host-derived endogenous ligands known as danger-associated molecular patterns. These endogenous danger response signals, such are uric acid and HMGB1, are released by stressed cells upon necrosis to promote an innate immune response through activation of PRRs (4, 5).

Toll-like receptors are the best-characterized PRRs. First identified in *Drosophila* (6), to date 11 separate receptors have been identified in humans, classified as TLR1–10, with human TLR11 believed to be a pseudogene (7). A further two TLRs, TLR12 and TLR13, have been described in mice, but are not found in humans. Although TLRs sense a broad range of ligands, derived from both exogenous microbial pathogens and host (damage-associated molecular patterns), they share a common structure. This consists of an extracellular ectodomain containing a variable number of leucine-rich repeat (LRR) motifs that mediate ligand binding, a single transmembrane helix, and an intracellular toll-like interleukin 1 receptor domain required for intracellular signaling (8). Dependent on which TLR is activated, specific adaptor molecules are recruited which can broadly be considered as MyD88 dependent or independent. It is this selectivity of adapter molecule recruitment that leads to the specificity of TLR signaling pathways and the subsequent inflammatory response. The role of TLR signaling in the pathogenesis of IBD, together with the potential for therapeutic modulation, is discussed in Section “Potential Therapeutic Targets Altering Signaling through TLRs.”

There is emerging evidence for the importance of another class of PRR, the CLR in IBD. CLRs comprise a large family of receptors that bind to carbohydrates *via* carbohydrate-recognition domains and appear of particular importance in mediating antifungal immunity, but are also able to recognize other pathogens including bacteria and protozoa (9). A polymorphism in the gene for Dectin-1 is linked to a severe form of ulcerative colitis, driven by an aberrant response to commensal intestinal fungi (10). Genetic variants in mannose-binding lectin (MBL) have been linked to Crohn’s disease (11) while mice deficient in MBL show increased susceptibility to experimental colitis (12). Macrophage galactose-type C-type lectin-1 and SIGN-R3 have also separately been linked to protective regulatory roles in murine models of colitis (13, 14). It is clear that CLRs may also act in synergy with TLRs, with a deficiency of both the CLR SIGN-R1 and TLR4 leading to reduced susceptibility to colitis in a murine model, with reduced responsiveness to the TLR4 ligand lipopolysaccharide (15).

## Overview of the NLR Family

The NLR family of proteins are cytosolic PRRs that sense a diverse range of microbial structures such as peptidoglycan and flagellin, and also endogenous danger signals, to trigger innate immune activation (16, 17). NLRs consist of three domains—an N-terminal protein interaction domain, a central NOD domain, and a C terminal LRR. The NLRs are divided into four subfamilies on the basis of their N-terminal effector domains: NLRA, acidic domain containing; NLRB, Baculovirus inhibitor of apoptosis protein repeat domain containing; NLRC, caspase recruitment domain (CARD) domain containing; NLRP, pyrin domain containing. NLRX represents other NLR proteins with no significant homology to the N-terminal domain of the other NLR subfamilies (18).

Functionally, the NLR family can be divided into further subgroups related to inflammasome assembly, autophagy, antigen presentation, signaling transduction, and transcription activation (19). The N-terminus effector domain that mediates protein–protein interactions is important in determining function, for example, the pyrin domain of the NLRP subfamily allows binding and activation of the caspase-1 inflammasome, while the CARD domain of the NLRC subfamily binds and activates receptor-interacting serine/threonine protein kinase 2 (RIPK2), activating downstream NF-κB, and MAPK signaling pathways (20, 21). The LRR domain is required for binding and detection of ligands and consists of leucine-rich amino acid strands forming a peptide loop.

The NOD domain, which has ATPase activity, is required for self-oligomerization of NLRs following binding of ligand to the LRR domain, likely facilitating binding and activation of downstream effector molecules *via* the N-terminus effector domain (22). The structural diversity of the LRR and N-terminus effector domains allows NLRs to interact with a wide array of ligands and binding partners, activating a broad range of signaling pathways. This breadth of role is reflected in the wide variety of human diseases that result from mutations in NLR-encoding genes (23).

## Regulation of NOD2 Signaling

Two related members of the NLR family, NOD1 and NOD2, are critical for the innate immune response to many bacterial infections. Both NOD1 and NOD2 respond to distinct structural motifs derived from intracellular peptidoglycan, a fragment of bacterial cell walls. NOD1 recognizes the dipeptide d-glutamyl-meso-diaminopimelic acid, present in all Gram-negative, and a limited number of Gram-positive bacteria such as *Listeria* spp. and *Bacillus* spp. (24, 25). By contrast, NOD2 recognizes muramyl dipeptide (MDP), the largest fraction of peptidoglycan consisting of one carbohydrate and two amino acids, present in all Gram-negative and Gram-positive bacteria (26). Activation of NOD1 and NOD2 signaling upon peptidoglycan sensing leads to their direct interaction with RIPK2 in a complex called “nodosome.” The nodosome formation leads to activation of pro-inflammatory and antimicrobial responses as discussed further in this review (27, 28). In 2001, *NOD2* was found to be associated with Crohn’s disease and it remains one of the strongest genetic risk factors (29, 30). Given this evidence, the review of innate mechanisms relating to NLRs will focus on NOD2.

NOD2 is expressed in cells of the gastrointestinal tract, specifically Paneth cells, intestinal epithelial cells (IECs), stem cells, and stromal cells. It is also expressed in the hematopoietic compartment in monocyte-derived cells including dendritic cells (DCs) and macrophages. In IECs, membrane targeting of NOD2 is required for NF-κB activation by MDP (31). NOD2 also recruits the autophagy protein autophagy-related 16-like 1 (ATG16L1) to the plasma membrane at the site of bacterial entry to drive autophagy (32). It remains unclear where NOD2 engages ligand in other cellular locations, such as ligands derived from endosome resident bacteria including *Salmonella enterica* serovar Typhimurium (33).

NOD2 consists of a LRR domain, a central NOD domain, and an N-terminal effector domain that contains two CARDs in tandem. Extrapolation of the recently solved crystal structure of NLRC4 predicts that the NOD domain is followed by a proximal helical domain, a winged-helix domain, and a distal helical domain (34). The ADP-mediated interaction between the NOD domain and winged-helix help stabilize a closed, auto-inhibited conformation, with the LRR occluding these two domains and maintaining a monomeric state (27, 34).

The precise mechanisms by which MDP enters cells and activates NOD2 remains uncertain, but a number of entry routes have been described: (1) phagocytosis of bacteria and degradation into fragments including MDP, (2) shedding of peptidoglycan by invasive bacteria (35), (3) uptake of peptidoglycan fragments from bacteria-derived extracellular outer membrane vesicles (36), (4) endocytosis (37), and (5) transport across host membranes through bacterial secretion systems or channels (27, 38). The relative importance of these varied mechanisms is likely to differ depending on the host cell type. Taken together, these mechanisms suggest that NOD2 may be able to sense extracellular bacteria, significantly expanding upon its classically described role as a sensor of invading cytosolic bacteria (33). The ability of soluble MDP to stimulate NOD2 *in vitro* further supports the presence of routes of direct entry for the ligand (27).

Following MDP binding and conformational change of NOD2 to an open structure and homo-oligomerization, NOD2 recruits RIPK2 *via* interaction between their CARD domains (27, 39). The NOD2–RIPK2 signaling axis has been extensively studied and mapped. RIPK2 is essential for NOD2 signaling, as evidenced by the failure of RIPK2-deficient murine macrophages to respond to MDP (40). Engagement of RIPK2 can occur *via* NOD1 or NOD2 but not *via* TLR-mediated signaling pathways (33).

Although it was initially uncertain whether MDP binds directly to NOD2 once it has reached the cytosol, recent reports support a direct interaction but do not exclude the requirement for accessory molecules (41, 42). It is generally believed that recognition is mediated by the LRR domain, although a potential role for the NOD domain has also been suggested (42). A mechanism has been proposed whereby ATP binding to NOD2 leads to homo-oligomerization and enhances MDP binding and subsequent signal transduction (42). It is likely that the distal helical domain is required to mediate NOD2 conformational change to an open activated structure upon ligand binding (34). However, further structural data are required to confirm the mechanism of NOD2 activation.

The stable ubiquitination of RIPK2 leads to the recruitment and activation of the kinase TAK1 (27). This activated TAK1 complex interacts with the simultaneously bound IκB kinase (IKK) complex, ubiquitinating and degrading IKKγ, which allows IKKα and IKKβ to phosphorylate IκKBα (NF-κB inhibitor IκB) (38). Phosphorylated IκKBα is degraded, allowing the translocation of NF-κB to the nucleus and inflammatory gene transcription (27, 43). In addition to NF-κB pathways, NOD2 activation *via* RIPK2/TAK1 also activates the MAPK regulated extracellular ERK1/2, JNK, and p38 (39). NOD2 can also activate p38 and JNK *via* CARD9 (44).

More recently, it has been shown that NOD2 is able to respond to pathogen-associated molecular patterns other than MDP, most notably viral ssRNA (45). This results in a quite separate RIPK2 independent signaling pathway, mediated by the mitochondrial antivirus signaling protein (MAVS). Engagement of MAVS at the mitochondria leads to a signaling cascade involving interferon regulatory factor 3 (IRF3) that produces interferon (IFN)-β (45). It was shown *in vitro* that NOD2 drives type 1 IFN production in response to a range of viruses containing an ssRNA genome including respiratory syncytial virus and influenza virus (45). This was confirmed *in vivo*, with NOD2 knockout mice demonstrating an increased susceptibility to infection with respiratory syncytial virus or influenza, although increased susceptibility to viral infections has not yet been shown in humans expressing NOD2 polymorphisms (45).

Much remains to be understood about how NOD2 signaling pathways are regulated but it is clear that a complex system of protein–protein interactions underlies this. In addition to the actions of the E3 ligases described above, roles for TRAF 2, 4, and 5 have been described (43, 46). NOD2 signaling can also be fine-tuned by the removal of ubiquitin. An important example is the action of ovarian tumor deubiquitinase A20, which inhibits NOD2 signaling by regulating the MDP-induced ubiquitination of RIPK2 (47).

Additional negative regulators include Erbin that directly binds to NOD2 and inhibits MDP signaling (48), Rac1 GTPase (49) and RIG-I (50). By contrast, GRIM-19 (51), a cell death protein, and CARD9 (44) positively regulate NOD2 signaling. It appears that the cytoskeleton plays a key role in modulating NOD2 activity; in addition to cytoskeletal-related proteins, Erbin and Rac1 GTPase, the intermediate protein filament vimentin is also important (52). Intriguingly, three RHO GTPases, including Rac1 GTPase, that play important roles in cytoskeletal modeling, are apparently able to activate NOD2 in the absence of MDP, raising the possibility of NOD2 stimulation in the absence of a bacterial pathogen (33, 53).

## The Interrelationship of NOD2 and TLRs

A complex interplay between PRRs provides cross-regulation of innate immune receptor signaling and can both amplify and suppress the immune response (54). The intricacy of this interrelationship results from the varied array of PRR ligands, many of which may arise from the same pathogen, and the divergent signaling pathways they may simultaneously induce (54). Synergistic signaling results from NOD2 acting together with a number of PRR-mediated pathways including NOD1, TLR2, TLR3, TLR4, and TLR9 to boost production of a range of both pro- and anti-inflammatory cytokines (IL-6, IL-8, TNF, IL-1β, and IL-10) in antigen-presenting cells (APCs) (22, 55–57). The cross-talk between NOD2 and TLR2 remains the most well characterized to date. Their close relationship is not surprising given that both respond to ligands derived from the same bacterial component, peptidoglycan. Although the precise mechanisms of cross-regulation are not well understood, both NOD2 and TLR2 activate separate upstream signaling cascades to recruit the same MAPK and NF-κB pathways, which play a central role in cytokine production (54). NOD2 and TLR2 also collaborate in adaptive immune roles, and they have been shown to cooperatively regulate the functional maturation of DCs (58).

## Mitochondria Provide a Structural HUB for Innate Immune Signaling

Mitochondria are highly active organelles that continuously relocate within the cell, undergoing fission, fusion, biogenesis, and mitophagy to maintain a pool suitably responsive to cellular demands. These processes are termed mitochondrial dynamics and control mitochondrial morphology, quality, abundance, and location, which are critical to the immune role of mitochondria (59). There is still much to understand about how the innate immune system regulates mitochondrial dynamics, but a recent study provides an interesting insight. Following recognition of bacteria by TLRs, macrophages were found to adapt their electron transport chain architecture by destabilizing complex I in a NLRP3 and reactive oxygen species (ROS)-dependent manner (60). This resulted in enhanced mitochondrial respiration from complex I and II and increased mitochondrial reactive oxygen species (mROS) and may regulate IL-1β and IL-10 production.

**Figure 1:**
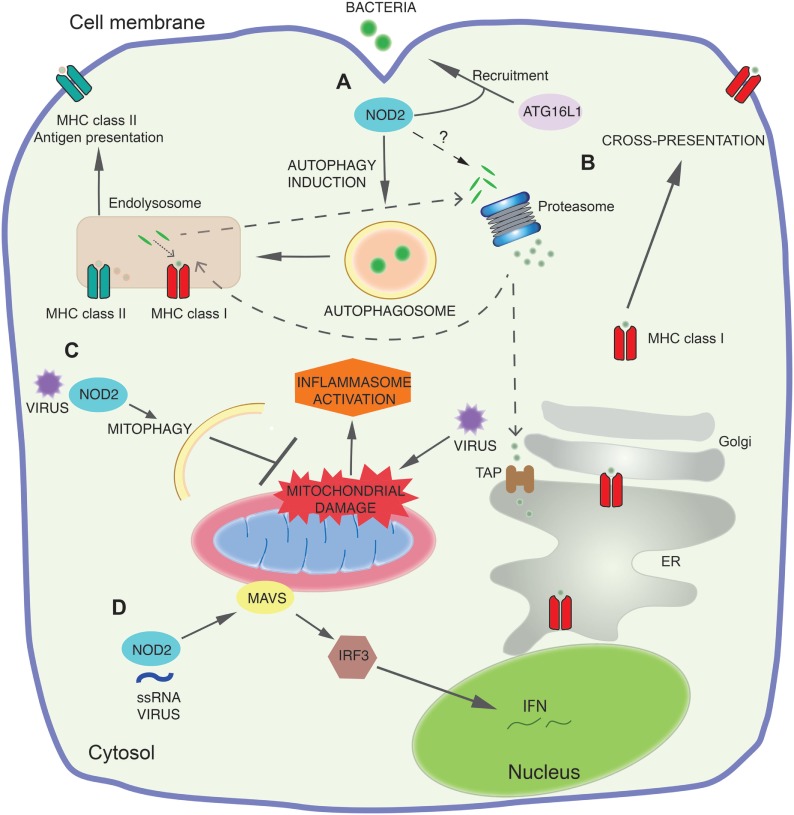
NOD2 signaling pathways. **(A)** At sites of bacterial entry to the cell cytosol, NOD2 recruits the autophagy protein autophagy-related 16-like 1 (ATG16L1) to the plasma membrane and together they direct autophagy of the invading bacteria. This leads to both direct bacterial killing and loading of the antigens to major histocompatibility complex class II (MHC II). **(B)** Early NOD2 engagement by components of bacteria increases cross-presentation for MHC class I-dependent antigen presentation. Antigens destined for cross-presentation can have different routes: they can be transported from the endocytic vesicles to the cytosol for proteasomal degradation and transported into the endoplasmic reticulum (ER) or back into the endosomal compartment for loading onto MHC class I. The molecular mechanisms by which NOD2 controls these pathways and proteasome function are still not defined. **(C)** NOD2 also functions as a viral pattern-recognition receptor and modulates immune response to viral infection. NOD2 drives RIPK2-mediated mitophagy of damaged mitochondria following viral invasion of the cytosol. This negatively regulates the NLRP3 inflammasome response by limiting the release of mitochondrial reactive oxygen species and other mitochondrial damage-associated molecular patterns following the mitochondrial damage caused by viral infection. **(D)** NOD2 recognizes viral ssRNA to trigger mitochondrial antivirus signaling protein (MAVS) mediated activation of interferon regulatory factor 3 (IRF3), leading to production of the antiviral interferon (IFN) response.

Mitochondria act as a platform for innate immune signaling, with key innate immune effectors assembling on the outer mitochondrial membrane (59, 61). One of the first proteins discovered was MAVS, a key adapter protein for the RLR signaling pathway that responds to viral infections (62). On viral infection and RLR triggering, MAVS is targeted to the outer membrane by a C-terminal transmembrane domain. Although it remains unclear precisely why MAVS must be recruited to the mitochondria, localization here is essential to mediate downstream signaling *via* NF-κB and IRF3 to regulate type-1 IFN production (61, 62). As described earlier, NOD2 can also engage MAVS in response to viral ssRNA triggering a similar IFN cascade (45). MAVS activation is additionally regulated by mitochondrial dynamics (63) and mROS (64) (Figure 1D). A further illustration of the intersecting nature of mitochondrial signaling pathways is the recent discovery of the regulation of the NLRP3 inflammasome by MAVS (65). Another mitochondrial membrane protein, cardiolipin, also controls NLRP3 inflammasome activation in an mROS-independent manner (66).

## Activation of the Immune Response Following Mitochondrial Damage

Given the ancient bacterial roots of mitochondria, it is unsurprising that mitochondrial injury releases compounds carrying bacterial molecular motifs, sensed as danger-associated molecular patterns, to trigger an immune response (67). Mitochondrial DNA (mtDNA), which shares hypomethylated CpG motifs with bacterial DNA, is one such danger-associated molecular pattern, which can be sensed by the innate immune receptor for CpG DNA, TLR9 (67). TLR9 activation leads to NF-κB and MAPK inflammatory cascades and can also signal through IRF7 to enhance type-1 IFN responses (68). mtDNA is also an important endogenous agonist for NLRP3 inflammasome (69), and in macrophages has been shown to activate the NLRC4 (70) and AIM2 (absent in melanoma 2) inflammasomes (69). Finally, mtDNA can also trigger pro-inflammatory cytokine responses *via* the cGAS PRR (71, 72). mtDNA-dependent inflammatory pathology has been described in a diverse range of diseases including heart failure, atherosclerosis, rheumatoid arthritis, and liver disease, and following bacterial infection (68).

It is essential that cells are able to efficiently eliminate damaged mitochondria and their danger-associated molecular patterns to prevent aberrant inflammation, and here autophagy plays a vital role. Mitophagy inhibits NLRP3 inflammasome activation by clearing damaged mitochondria and limiting the release of both mROS and mtDNA (69, 73). Mice deficient in the autophagy protein LC3B showed enhanced caspase-1-dependent cytokines in sepsis models (69). As described earlier, following viral infection the NOD2–RIPK2 axis also acts to increase mitophagy and limit inflammasome activation (Figure 1C). These studies underline the importance of mitophagy in responding to the mitochondrial damage that occurs during an immune response, thereby preventing hyperactivation of the immune system.

## Mitochondria and Crohn’s Disease

While it is clear that mitochondria are of central importance in immunity, their role in Crohn’s disease is not well understood. A number of studies suggest the presence of mitochondrial dysfunction in IBD. Enterocytes isolated from patients with IBD have abnormal mitochondrial morphology (74), and murine models of colitis result in similar mitochondrial changes in IECs (75). There is evidence of elevated ROS, although the source was not clarified, and impaired mitochondrial membrane potential in patients with active Crohn’s, which was found to improve on disease remission (76). In pediatric Crohn’s disease patients, proteomic analysis of mucosal biopsies suggested a marked downregulation of mitochondrial proteins, including components of the mitochondrial respiratory chain (77). It has been suggested that IBD is a state of energy deficiency, with impaired mitochondrial β-oxidation of fatty acids implicated (78). This theory is supported by the findings that, mutations in *SLC22A5* (*OCTN2*), which encodes a carnitine transporter, are associated with Crohn’s disease (79), with carnitine essential for fatty acid oxidation. Mice lacking *OCTN2* develop spontaneous intestinal inflammation and atrophy (80). Conversely, mice carrying mutations that increase mitochondrial oxidative phosphorylation activity and ATP generation are protected from chemical models of colitis (81). It is clear that more research is needed to elucidate the contribution of mitochondrial dysfunction to Crohn’s disease, but the potential intersection with processes such as autophagy and inflammasome activation is intriguing.

## Innate Immune Modulation of Autophagy

A distinct role for innate immunity is the induction of a form of autophagy, macroautophagy (32, 82), a highly conserved mechanism for the bulk degradation of cellular contents. Macroautophagy is distinguished from the two other primary types of autophagy in mammalian cells, microautophagy and chaperone-mediated autophagy, by the formation of a double membrane bound phagophore and autophagosome (83). In its most basic form, autophagy represents a cellular adaptation to starvation and allows the non-specific breakdown of a cell’s own constituents to recycle nutrients and balance biosynthetic pathways (84, 85). This form of autophagy, known as non-selective autophagy, is also induced by cellular stress. However, autophagy can also be utilized by cells in a more precisely targeted process (selective autophagy) for various indications, and indeed is not restricted to “self-constituents” as it can target invading pathogens (86). This important host defense against pathogens, termed xenophagy, can target a range of invasive bacteria including *S. enterica* serovar Typhimurium, *Listeria monocytogenes, Shigella flexneri*, and *Mycobacterium tuberculosis*, as well as bacteria internalized by phagocytosis (87). Both NOD2 and TLRs can mediate xenophagy and this influences loading of microbial antigens to late endosomal compartments, where substrates are then degraded by lysosomal hydrolases. During antigen presentation, foreign proteins are captured by autophagosomes and are delivered to major histocompatibility complex (MHC) class II-processing and -loading compartments (Figure 1A).

Following induction of autophagy, the core pathway of macroautophagy begins with the nucleation of a double-membrane phagophore. The origin of the phagophore is a source of considerable debate, with a role proposed for numerous membrane compartments including the endoplasmic reticulum (ER) and the mitochondria (88, 89). This step and the subsequent extension of the phagophore into an autophagosome that engulfs the targeted cytoplasmic components requires the autophagy ubiquitin-like protein LC3 (the mammalian homolog of ATG8) (90). To date, over 40 proteins have been identified as important for macroautophagy, primarily the autophagy-related genes first mapped in yeast (91) with mammalian homologs subsequently described. Broadly, they can be grouped according the stage of autophagy at which they function—(1) induction, (2) nucleation and expansion, (3) fusion, and (4) degradation and efflux (90). A number of these proteins are involved in the processing of LC3, underlining its importance. The ATG4 protease first cleaves the inactive pro-form of LC3 at its C-terminus to generate the cytosolic LC3-I. LC3-I is then conjugated to phosphatidylethanolamine in the phagophore membrane by ATG3 and 7, producing the lipidated LC3-II, which initiates the formation and maturation of the autophagosome. A critical role of LC3 is to recruit other autophagy proteins to the autophagosome, including the ULK1 kinase (90, 92) and ATG13 (90). This typically requires an LC3-interacting region (LIR), characterized by a WXXI/L sequence, with tryptophan (W) beginning the sequence, any two amino acids (XX) following, and either isoleucine (I) or leucine (L) two residues downstream (93). A number of LIR containing autophagy proteins also contain an ubiquitin-binding domain, allowing recruitment of the aforementioned ubiquitinated targets to the autophagosome. Important examples in xenophagy are p62 (SQSTM1) (94), NDP52 (95), NBR1 (96), and optineurin (OPTN) (97). In addition, and vital to the concept of xenophagy, an LC3-decorated single membrane phagosome may also become sequestered within an autophagosome (98). This type of xenophagy is restricted to phagocytic cells (macrophages, DCs, and neutrophils).

During the process of autophagosome maturation, LC3-II is deconjugated by ATG4 and thus released from the outer membrane. By contrast, the GATE-16/γ-aminobutyric acid receptor-associated family (GABARAPs) appear important for the maturation of the autophagosome (99). Following closure and maturation, the autophagosome progresses toward fusion with the lysosome. The autophagosome may fuse with a late endosome forming an amphisome (100), which subsequently fuses with a lysosome to generate an autolysosome. Alternatively, an autophagosome may fuse directly with a lysosome, and this should also be termed an autolysosome (101). By contrast, when a phagosome is incorporated into an autophagosome and fuses with a lysosome, this should be distinguished as an autophagolysosome (101). The term autophagolysosome also describes the fusion of an LC-decorated phagosome with a lysosome (102), a process independent of autophagy termed LC3-mediated phagocytosis, but which shares a number of overlapping features with autophagy.

Three protein families are key to the process of autophagosome–lysosome fusion. Rab GTPases localize to the membranes of both structures, recruiting membrane-tethering complexes that bridge the autophagosome to the lysosome. Soluble *N*-ethylmaleimide-sensitive factor attachment protein receptors then drive the fusion of the opposing lipid bilayer membranes (103). In addition, GABARAPs also regulate modulate autophagosome–lysosome fusion by regulating the generation of phosphatidylinositol-4-phosphate (104). Once fused, lysosomal hydrolases digest the contents of the autophagolysosome.

## Innate Immunity Affects the Unfolded Protein Response (UPR)

A cascade of intracellular pathways have evolved to respond to protein misfolding—this is known as the UPR (105). The combined action of three ER transmembrane stress sensors is responsible for UPR activation: inositol-requiring enzyme 1α (IRE1α), PKR-like ER kinase (PERK), and activating transcription factor 6α (ATF6α). Usually, the luminal domains of these proteins are inactive through association with binding immunoglobulin protein (BiP). However, BiP has higher affinity for misfolded proteins. Therefore when misfolded proteins accumulate, BiP dissociates from the stress sensors activating downstream signaling cascades (106). These signaling pathways lead primarily to a reduction in the quantity of proteins that enter the ER. Also, through increased transcription of ERAD- and autophagy-related proteins, misfolded proteins are eliminated. Finally, the ER expands, and the capacity to refold proteins is increased (107–110).

Dimerization of IRE1α, followed by release from BiP, initiates splicing of a single mRNA encoding X-box-binding protein 1 (XBP1) (111, 112). This generates XBP1s, an activator of transcription, which induces the transcription of chaperones and protein-folding enzymes resident in the ER. Together, these increase ER size and function (113). Dissociation of BiP from PERK allows PERK homodimerization and autophosphorylation to activate the cytoplasmic kinase domain. Activated PERK attenuates global translation of mRNA by inhibiting eIF2-TC to enable cells to temporarily manage ER stress (114, 115). Finally, following BiP dissociation, ATF6α relocates to the Golgi apparatus. Here, it is processed by site 1 and 2 proteases (S1P and S2P) producing a p50 fragment. This fragment translocates to the nucleus and induces gene expression related to proteins that increase overall ER capacity, the ability to refold proteins, and activates the ERAD pathway (116–118).

Inflammation represents a critical factor in the induction of the UPR. Immune cells are highly sensitive to environmental factors which induce ER stress. Protein-folding demand is markedly increased following pathogen exposure (105). ER stress and TLR signaling are linked—TLR signaling in macrophages induces ER stress and ER stress acts to increase the response to TLR signaling. The TLR 2 and 4 ligands, Pam3CSK4 and LPS, induce IRE1α activation. IRE1α-induced XBP1 splicing in response to TLR ligation affects pro-inflammatory cytokine production, such as the production of IL-6, TNF, and IFNβ. Increased IL-1β production results from the activation of glycogen synthase 3β (GSK3β) which is IRE1α dependent. Furthermore, GSK3β inhibits ongoing splicing of XBP1, attenuating TNF transcription thereby altering the inflammatory response (119, 120).

Dysregulation in UPR signaling have been associated with various complex inflammatory diseases, including IBD. Among the risk genes associated with IBD identified by genome-wide association studies (GWAS), some encode for proteostatic proteins including Orosomucoid-like 3 (*ORMDL3*) or anterior gradient 2 (*AGR2*) (121–124). Paneth cells are abnormally located in the ileum of *Agr2*-deficient mice who also exhibit reduced mucin 2 (MUC2) expression in goblet cells. These mice also develop spontaneous ileo-colitis and an activated UPR (125). Mice with a knockout of XBP1 specifically in IECs (*Xbp1Δ*^IEC^) exhibit signs of ER stress. They are more susceptible to chemical colitis induced by dextran sulfate sodium (DSS) and develop a spontaneous ileitis (126). If these mice also lack the autophagy gene ATG16L1 in IECs they develop a Crohn-like transmural spontaneous enteritis (127). Importantly, patients carrying the Crohn’s risk allele ATG16L1 (T300A) have evidence of ER stress in Paneth cells (128).

## PRRs Influence Adaptive Immune Responses

Activation of the innate immune signaling pathways through PRRs discussed above provides immediate detection of microbial presence and viability, which is necessary to determine a successful activation of naïve T cells and generate appropriate effector responses. Activation of PRRs leads to a significant change in the phenotype of APCs, which is characterized by enhanced expression of costimulatory molecules and increased secretion of pro-inflammatory cytokines (129). In addition, signals derived from PRRs in DCs determine whether the antigen presentation machinery leads to activation or cross-tolerance of T cells, depending on whether or not DCs are exposed to PRR ligands and on the length of this exposure (130). This contributes, in physiological conditions, to minimize the risk of generating pro-inflammatory responses to self-antigens, to maximize T-cell priming against microbial antigens during the initial phase of DC maturation, and to temporally control MHC class I and II antigen presentation and prevent excessive priming during chronic phases of pathogen handling. Consequently, dysregulation of these mechanisms may be highly relevant in the pathogenesis of IBD.

## NOD2-Dependent Generation of CD4^+^ T Cell Responses

NOD2 recruits the autophagy protein ATG16L1 to the plasma membrane at the site of bacterial entry to direct autophagy. This facilitates bacterial trafficking to the autophagosome through a signaling pathway independent of RIPK2. NOD2 signaling *via* RIPK2 also upregulates the autophagosome formation and increases autophagic flux, further potentiating autophagy. This is required for the generation of MHC class II antigen-specific CD4^+^ T cell responses (82) (Figure 1A). Importantly, DCs expressing the Crohn’s disease NOD2 and ATG16L1 variants have reduced autophagic response and MHC class II antigen presentation in response to MDP. Decreased macroautophagy due to NOD2 and ATG16L1 mutations may impair innate resistance to invading bacteria and, thereby, trigger inflammation as a result of increased antigenic load or lead to insufficient tolerance induction against commensals in the gut and trigger Crohn’s disease (82).

In the context of NOD2-dependent generation of MHC class II antigen-specific CD4^+^ T cell responses, several studies also suggest that NOD2 signaling influences CD4-specific adaptive immune responses. The induction of IL-17 by bacteria-primed DCs is not through the TGF-β–IL-6 pathway in naïve human Th cells. Instead, Th17 cells develop from memory Th cells (27). In addition, MDP programs DCs to increase IL-23 and IL-1 production which orchestrates Th17-mediated immunity in humans. In line with this, expression of IL-17 following MDP stimulation is impaired in DCs derived from Crohn’s disease patients with polymorphisms in the NOD2 gene. This can be attributed to loss of NOD2/TLR synergy on production of IL-1α and IL-1β, and IL-23 (131, 132).

It is important that the synergistic effects of NOD2 with other TLR signaling pathways inducing effector cytokines such as IL-23 are tightly regulated so that homeostasis can be restored at the termination of an immune response. MicroRNAs (miRNAs) are important regulators of gene expression whose main function is to repress target mRNA levels including regulators of innate immune responses by targeting key signaling proteins and cytokines (133–137). Our group has previously demonstrated that NOD2 can regulate induction of the miRNA family 29a, 29b, and 29c and induces this family alone or additively with TLR2 or TLR5 in DCs. miRNA-29 downregulates IL-12p40/IL-23 and attenuates Th17 CD4^+^ T cell responses; however, Crohn’s disease DCs expressing associated NOD2 variants are incapable of inducing miR-29 following NOD2 triggering. Therefore, NOD2 has an immunoregulatory function in human DCs of the miR-29 family, and a loss of miR-29 induction in Crohn’s DCs might contribute to the abnormal elevation of IL-23 observed in inflamed lesions during this disease (132).

NOD2 activation by MDP in mice generates specific Th2-type immune responses. Costimulation with TLR agonists promotes the priming of not only Th1 and Th2 but also Th17 responses (138, 139). In this context, direct triggering of NOD2 by MDP, generates a specific Th2-type immune response and stromal NOD2 expression is needed to prime effector CD4^+^ Th2 responses. In addition, NOD2-dependent stimulation induces OX40 ligand, necessary for Th2 immunity (140, 141). A more recent study has investigated the effect of *Nod2* deletion in a spontaneous mouse model of chronic intestinal inflammation, SAMP1/YitFc, characterized by a progressive cobblestone CD-like ileitis that develops in the absence of chemical, genetic, or immunological manipulation (142). *Nod2* deletion in SAMP1/YitFc mice was associated with inhibition of the Th2 cytokines IL-4, IL-5, and IL-13 whereas no effect was observed in Th1 cytokine expression, including TNF-α and IFN-γ. In addition, the role of Th2 effector signaling pathways were also affected by NOD2 deletion, with decreased phosphorylated-STAT6 and GATA3 in the gut mucosa of SAMP × NOD2^−/−^ mice. These effects were observed in the presence of the same bacterial flora, which suggests that changes in the bacterial community are not associated with the effects of NOD2 in ameliorating intestinal inflammation in SAMP1/YitFc mice, and that NOD2 regulates Th2 responses in the intestine independent of acute dysbiosis (142).

With the availability of new drug compounds that target inhibition of NOD2 and RIPK2 signaling, potential pharmacological inhibition of NOD2 signaling may be a reasonable therapeutic strategy to prevent Th2-driven intestinal inflammation and other CD4 T cell responses.

## Innate Immune Regulation of Cross-Presentation and CD8^+^ T Cells Responses

Cross-presentation represents a critical mechanism for priming adaptive immune responses against exogenous antigens derived from microbial pathogens or tumors (143), which are presented by MHC class I molecules (144). During cross-presentation, the establishment of CD8^+^ T-cell-mediated responses is dictated by DCs whose function is to acquire exogenous antigens and direct the formation of a complex between the MHC-I peptide and a cognate TCR leading to activation and proliferation of antigen-specific CD8^+^ T cells. Different pathways and subcellular locations regulating cross-presentation have been described, including the vacuolar and phagocytic pathways (143). Engagement of the vacuolar pathway implicates the degradation of antigens by endosomal or phagosomal proteases (i.e., cathepsin S) and the resultant peptides are loaded onto MHC class I molecules (145). By contrast, during activation of the phagocytic pathway, internalized antigens from endosomes or phagosomes are exported to the cytosol where they are degraded by the proteasome. The resultant peptides are then transported back into the phagosome and loaded onto MHC-I (144, 146) or, instead, peptides are transported *via* TAP into the ER, for loading onto ER-resident heavy chain-B2m complexes (147) (Figure 1B). It has been indicated in several studies that PRRs signaling influence on multiple processes associated with cross-presentation. Thus, any dysfunction of these mechanisms may lead to abnormal activation of CD8^+^ T cells responses associated with inflammation.

There is temporal control of MHC-I antigen presentation by the innate immune system, with initial PRRs engagement promoting its efficiency during the early or acute phase of microbial exposure and with prolonged stimulation leading to mechanisms to prevent excessive priming during chronic phases of pathogen handling. Following a short stimulation of TLR4 by LPS in DCs, antigen translocation from the phagosome to the cytosol is increased. During this phase, Blander and colleagues have also recently shown that the recruitment to phagosomes of MHC class I molecules, stored in endosomal recycling compartments (ERCs), is enhanced by TLR4 stimulation (148). In fact, LPS stimulation leads to IKK2-dependent phosphorylation of phagosome-associated SNAP-23 (synaptosome-associated protein of 23 kDa) promoting fusion between ERCs and phagosomes. This mechanism acts to deliver to phagosomes enough numbers of MHC-I molecules once TLRs senses microbial components to increase cross-presentation and priming of CD8^+^ T cells (148, 149).

During the intermediate phase of DC maturation followed by 3–16 h of TLR engagement, the efficacy of cross-presentation is still increased (150, 151). During this phase, DCs exhibit increased endocytosis, proteasomal and TAP activity, delayed phagosomal degradation, and decreased acidification (mediated by a decrease in recruitment of lysosomal proteases to phagosomes). RAB34-dependent peri-nuclear clustering of lysosomes and reduced shift of phagosomes along microtubules prevents their fusion, with the end result of increasing the efficiency of cross-presentation (152, 153).

During the late stage of DCs stimulation by TLR agonists (24–40 h), there is a markedly reduced efficiency of cross-presentation (150). This is likely due to decreased antigen uptake or antigen export to the cytosol (154, 155).

Given the presence of NLRs at sites that are in close proximity to phagosomal and endosomal membranes containing high levels of bacterial components, engagement of their signaling pathways may play a critical role in interacting with the MHC class I antigen presentation machinery. However, there are only few studies investigating how NLR activity affects cross-presentation (156).

NLRP3 inflammasome and caspase-1 activation modulate phagosome activity by causing locally restricted modification of the proteins associated to the organelle, which results in induction of the microbicidal activity (157). In fact, phagosome-associated caspase-1 can control the activity of NADPH oxidase NOX2 inducing changes on pH of the vacuole. In line with this, NLRP3-deficient and caspase-1-deficient cells fail to induce phagosome acidification in response to microbial infection (157). Since cross-presentation of phagocytosed antigens to CD8^+^ T cells occurs primarily from a non-acidified phagosome, activation of the inflammasome negatively impact cross-presentation by controlling the pH of phagosomes, which accelerates degradation of antigens.

Early stimulation of both NOD1 and NOD2 signaling alone or together with other PRRs enhances cross-presentation while during the late phase DC maturation their stimulation lead to decreased cross-presentation. NOD1 and NOD2 activation by peptidoglycan in DCs increase cross-presentation (Figure 1A) *via* upregulation of intracellular components, such as TAP, SEC61, and calnexin, which are essential for MHC class I-dependent antigen presentation and enhanced antigen-specific CD8^+^ T cells. During this process, NOD/RIPK2-mediated signals might mimic the TLR4–MyD88 signals necessary to induce recruitment of TAP to the early endosomes, which is an important step for enhancing cross-presentation of soluble antigens (158). By contrast, peptidoglycan pre-treatment of DCs led to progressive inhibition of cross-presentation over time, with decreased cross-presentation after 12 h and complete inhibition after 18 h (159). This demonstrates that maturation of DCs by NOD1 and NOD2 engagement, prior to antigen encounter negatively modulates cross-presentation. Therefore, NOD-dependent signaling pathways temporally control MHC class I antigen presentation and may prevent excessive priming during chronic phases of pathogen handling. This may be highly relevant in the context of chronic intestinal inflammation in which loss of function associated polymorphisms in the NOD2 gene are associated with increased susceptibility to Crohn’s disease. As increasing evidence suggests that CD8^+^ T cells may play an earlier role in IBD development than the CD4^+^ T cells (160), whether dysregulated NOD2 signaling may affect CD8^+^ T cell responses by aberrant priming *via* the cross-presentation pathway remains to be investigated.

## Modification of PRR Signaling in the Management of Intestinal Inflammation

### Modulating NLR Pathways—NOD1, NOD2, and NLRP3

NOD1, polymorphisms of which are associated with human IBD (161), is constitutively expressed in IECs and has been shown to activate NF-κB by pathogens in particular those which have developed methods to bypass sensing by TLRs (162). Furthermore, NOD1 is required for innate immune responses in human IECs to *Campylobacter jejuni* infection and transient knockdown of NOD1 increases bacterial invasion (163). However, NOD1^−/−^ mice do not exhibit any pathological differences to wild-type in a *Salmonella*-induced colitis model, whereas NOD1^−/−^NOD2^−/−^ double knockout resulted in milder colitis (164). Contrary to this, NOD1 deficiency has been shown to result in increased colitis-associated colonic cancer in a chemical colitis model (165), which may, in part, reflect differences in colitis modeling. Together, this suggests that augmentation rather than complete inhibition of NOD1 may derive benefit in intestinal inflammation. There are currently no preclinical or clinical studies modulating NOD1 signaling directly although a number of inhibitors have been developed, including Noditinib-1 (ML130) and ML146, to enable further study of this pathway (166–169).

The function of NLRs in intestinal inflammation is of particular interest given the strong association between NOD2 polymorphisms [R702W, G908R, and 1007fs (3020insC) (29, 30)] and Crohn’s disease. Patients with a NOD2 variant are more likely to have disease affecting the terminal ileum, exhibit a fibrostenosing phenotype, and to require surgical intervention (170). Interestingly, there are no differences in phenotype between homozygotes or compound heterozygotes for the three polymorphisms listed. Approximately half of patients with Crohn’s disease have at least one mutation in NOD2 (170) and importantly not everyone in the wider population with NOD2 variants develop intestinal inflammation (171, 172) highlighting the complexities of this disease and the non-essential role of NOD2 in disease pathogenesis. Unlike NOD1, NOD2 is expressed predominantly in myeloid cells (173). However, a role for recognition of intracellular bacteria in IECs, which is lost in 3020insC mutants, has been demonstrated (174).

Given that NOD2 polymorphisms are generally thought to lead to loss of function, it is intriguing as to why these might lead to an increased risk of intestinal inflammation. It is unclear as to whether repair of NOD2 signaling defects might represent a therapeutic strategy for patients expressing Crohn’s disease-associated polymorphisms (175, 176). An alternative to targeting the receptors directly is modulating the function of downstream signaling mediators such as RIPK2. RIPK2 inhibitors have shown benefit in inflammatory disease including in the SAMP1/YitFc model of Crohn’s disease-like ileitis (177–179) although there are no clinical studies as yet in intestinal inflammation.

Polymorphisms in the NLRP3 gene are associated with Crohn’s disease (180) although the effect size of this may be small (181). Mice deficient in NLRP3 are more susceptible to chemical colitis through lack of IL-18 (182) but, in contrast, excessive IL-1β and IL-18 production is seen in human IBD tissues suggesting activation of NLRP3 may mediate chronic intestinal inflammation [discussed in Ref. (123)]. To this end, inhibitors of the NLRP3 inflammasome such as *Astragalus polysaccharide* (183, 184), INF39 (an acrylate derivative) (185), and levornidazole (186) have been shown in experimental models to improve colitis. The peptide SPA4 has anti-inflammatory actions through indirect suppression of the NLRP3 inflammasome by interacting with TLR4 (187) although this has not yet been used in colitis models. However, the plant flavonoid alpinetin does improve DSS colitis through a similar mechanism of modulation of the NLRP3 and TLR4 pathways (188). Recently, a novel inhibitor of NLRP3, CY-09, was identified which binds to the NACHT domain of the complex to inhibit activation in murine macrophages and human PBMCs, and in a relevant murine model of NLRP3-associated inflammation (189). Ongoing study is required to elucidate whether this compound is of benefit in intestinal inflammation. Interfering with inflammasome-related cytokines may hold promise for the management of human IBD. A UK-based, multicentre, randomized, double-blind, placebo-controlled trial of the IL-1 receptor antagonist Anakinra alongside corticosteroids in acute severe colitis is planned.^1^ A comprehensive review of the role of the NLRP3 inflammasome in intestinal health and disease has recently been published (190).

### Potential Therapeutic Targets Altering Signaling through TLRs

#### TLR2

TLR2 has been shown to be essential for tight junction function in the intestine through MyD88 and PI3K/Akt (191). In this study, mice deficient for TLR2 or MyD88 demonstrated tight junction dysfunction when challenged with DSS while oral treatment with a TLR2 ligand, Pam3CSK4, improved barrier function and reduced colonic inflammation. Another group has shown that deletion of TLR2 in the multidrug resistance model of colitis resulted in more aggressive disease (192). The commensal microbiota may yield benefits for modulating inflammation through TLR2. Polysaccharide A (PSA) is produced by the mammalian intestinal commensal *Bacteroides fragilis*. PSA acts *via* TLR2 on APCs to modulate Th1/Th2 balance (193) and *via* plasmacytoid DCs to induce regulatory T cells (194). In a *Helicobacter hepaticus* model of colitis, co-colonization with *B. fragilis* resulted in a milder disease (195). Similar effects were seen with purified PSA alone (195). Oral PSA was of benefit in a 2,4,6-trinitrobenzenesulphonic acid (TNBS) chemical colitis model through increased production of the anti-inflammatory cytokine IL-10 (195). These effects on IL-10 levels have been replicated in germ-free mice challenged with DSS. In those mice which had prior administration of *B. fragilis*, this resulted in improved histology, reduced mortality, reduced pro-inflammatory TNF-α mRNA, and increased IL-10 mRNA (196). Surprisingly, despite the improved colitis, a significant increase in colonic pro-inflammatory IL-17 mRNA was seen in the *Bacteroides* group. A subsequent study by the same group confirmed the essential role of TLR2 in the modulation of colitis by *B. fragilis*. TLR2^−/−^ mice did not gain appreciable benefit from *Bacteroides* administration as measured by colon length and histological scores, and these mice also fail to upregulate IL-10 transcription following bacterial administration (197). Similar to the findings for PSA and *B. fragilis*, curli fibers (amyloid fibers in enteric biofilms) are recognized by TLR2, result in IL-10 production, and ameliorate TNBS-induced colitis (198).

Other avenues have corroborated the role of TLR2 in mediating inflammation. The TLR2 agonists lipoarabinomannan and lipoteichoic acid reduced indomethacin-induced murine ileitis *via* modulation of TLR4 pathways on macrophages and effects on leukocyte migration (199). The lipoxin A4 agonist, BML-111, alters the expression of TLR2 and 4 in a murine cecal ligation/puncture model of sepsis with improvements in pro-inflammatory IL-6 and TNF-α production (200). In addition to directly acting agonists, interfering with TLR2 dimerization has been shown to ameliorate DSS colitis through effects on monocyte activation (201) and to improve Pam3CSK4-induced hepatic inflammation (202). VB-201, an oxidized phospholipid mimic which is orally available, binds to TLR2 and CD14 to limit downstream inflammatory pathways and has beneficial effects on atherosclerosis (203) and experimental autoimmune encephalomyelitis (204). In addition to directly modulating TLR signaling pathways, effects are mediated through inhibition of monocyte migration (205). A phase 2 study of VB-201 in mild-to-moderate ulcerative colitis has been completed but the results have not yet been published (NCT01839214). A humanized IgG4-monoclonal anti-TLR2 antibody (OPN-305) is effective in a porcine model of cardiac ischemia–reperfusion injury (206) and has been shown to be well tolerated and effective in reducing IL-6 production from peripheral whole blood in a phase 1, randomized, double-blind, placebo-controlled clinical trial (207).

#### TLR3

TLR3, which responds to viral double-stranded RNA and is the only TLR not to signal through MyD88 (208), is expressed in human IBD tissues and stimulates the production of the antimicrobial peptide lipocalin-2 in the HT29 colonic epithelial cell line (209). Activation of TLR3 using poly(I:C) has been shown to ameliorate DSS colitis (210) through maintenance of epithelial barrier integrity (211). The enteric virome and an intact TLR3 signaling pathway is important in maintaining intestinal health. Treatment of wild-type mice with antiviral agents before administration of DSS results in worsened colitis, similar to TLR3^−/−^TLR7^−/−^ double knockout, and this is probably mediated through effects on IFNβ production by plasmacytoid DCs (212). Preconditioning of human umbilical cord-derived mesenchymal stem cells with poly(I:C) enhanced the immunosuppressive effects in both TNBS (213) and DSS (214) models of colitis. Although not yet used in human IBD, the TLR3 agonist rintatolimod has benefits in chronic fatigue syndrome/myalgic encephalomyelitis (215, 216), a disease of uncertain etiology but with evidence of viral triggers to the disease and impaired natural killer cell function (217).

Poly(I:C) is rapidly hydrolyzed in serum but can be stabilized with poly-l-lysine and carboxymethylcellulose (polyICLC) to resist this (218). PolyICLC is under evaluation as part of cancer therapy as a vaccine adjunct and may have roles in modulating cross-presentation of antigens by APCs to naïve CD8^+^ lymphocytes [reviewed in Ref. (219)]. Although not yet used in models of intestinal inflammation, polyICLC is protective in models of infections caused by influenza (220) or Dengue virus (221), murine cryptococcosis (222), and has been shown to enhance T cell responses in the lung of non-human primates when coadministered with anti-CD40 (223). An alternative to PolyICLC is PIKA, a stabilized double-stranded RNA, which has been shown to promote the maturation of DCs (224) but has been studied less extensively.

#### TLR4

In addition to the studies on SPA4 (187), alpinetin (188), and BML-111 (200) detailed above, other lines of enquiry suggest that modulation of TLR4 signaling may yield benefit in the management of intestinal inflammation. Unlike studies on TLR3 where enhanced signaling is effective the evidence with TLR4 is predominantly in blocking signaling. An *in vitro* study using SPA4 demonstrated a reduction in NF-κB-dependent cytokine production, migration, and invasion of the SW480 colonic cancer cell line suggesting that interfering with TLR4 signaling may be of benefit in colitis-associated cancer (225). To this end, transgenic mice with constitutively activated TLR4 are more susceptible to both DSS colitis and colitis-induced neoplasia (226) and the TLR4 antagonist 1A6 inhibits neoplasia in this model (226). Previously, it had been shown that 1A6 improved DSS colitis, but not the adoptive transfer model of colitis, although adverse effects on mucosal healing were noted (227). An alternative TLR4 antagonist, CRX-526, blocks the ability of LPS, the natural ligand for TLR4 (208), to induce pro-inflammatory cytokines and improves both DSS and multidrug resistance gene 1a-deficient models of colitis (228).

Infection of rhesus macaques with *Shigella dysenteriae* caused colitis, which was inhibited by oral administration of a small, non-absorbable polypropyletherimine dendrimer glucosamine (229), which acts to inhibit LPS signaling through TLR4–MD2 (230, 231).

The small molecule, TAK-242 (resatorvid), binds to the intracellular domain of TLR4 to inhibit signaling by interfering with the interaction between TLR4 and its adaptor molecules to inhibit NF-κB activation and interleukin-1 receptor-associated kinase (232). Two phase 3 clinical trials of resatorvid in severe sepsis have been performed (NCT00143611 and NCT00633477). Unfortunately, neither trial has been published nor the product was discontinued by the company leaving open the question as to its efficacy in intestinal inflammation.

Alkaline phosphatase has been shown to detoxify LPS by dephosphorylation of the lipid A component (233–235). The potential benefit of alkaline phosphatase in inflammation was demonstrated in mice and piglets challenged with LPS (236) and in zebrafish was shown to be important in mucosal immunity to gut microbiota (237). In patients with IBD (both ulcerative colitis and Crohn’s disease), there is reduced expression of alkaline phosphatase in both the inflamed and non-inflamed epithelium (238). Furthermore, oral administration of acid-resistant alkaline phosphatase (to prevent degradation in the stomach) to rats undergoing DSS challenge ameliorated colitis (238). An uncontrolled phase 2 trial of intraduodenal alkaline phosphatase in patients with ulcerative colitis demonstrated reductions in C-reactive protein, fecal calprotectin, and clinical activity indices with no particular safety concerns identified (239). Thus, modification of TLR4 signaling through detoxifying its primary ligand is promising in intestinal inflammation, and this has been the subject of a recent review article (240).

Contrary to the beneficial effects of the pharmacological inhibitors on TLR4-induced inflammation, there is some evidence, related to the pediatric condition necrotizing enterocolitis, that TLR4 signaling may be essential for controlling inflammation. Use of probiotic-conditioned media from *Bifidobacterium longum* subsp infantis prevents IL-6 induction in immature enterocytes but requires an intact TLR4 signaling pathway through TLR4–IRAK1–AP1 (241). Therefore, TLR4 inhibition may not be of benefit in all types of intestinal inflammation. A phase 2 clinical trial of *Bifidobacterium infantis 35624* in maintenance of remission in ulcerative colitis was registered in 2007 but the outcome is not known (NCT00510978). However, a different strain of this bacterium (strain 24737) is a component of the probiotic VSL#3 which has been shown in a recent systematic review to be of benefit in inducing remission in ulcerative colitis (242).

Altering TLR4 expression has also shown promise for understanding and modulating intestinal inflammation. The colons of mice deficient in corticotropin-releasing factor express less TLR4 mRNA and develop a more severe colitis in response to DSS (243). The flavonoid Baicalin improves colitis to a similar degree as mesalazine with a reduction in colonic TLR4 as measured by immunohistochemistry and a reduction in NF-κB-dependent pro-inflammatory cytokine production (244). TLR4 expression is reduced in enteric glial cells by the endocannabinoid-related lipid ligand palmitoylethanolamine, in a PPARα-dependent manner, to inhibit NF-κB activation, which is associated with a reduction in severity of DSS colitis (245).

Although no clinical studies of TLR4 modulation in intestinal inflammation have yet been performed, the TLR4 antagonist eritoran tetrasodium (E5564) has been used in a phase 3, randomized, double-blind, placebo-controlled clinical trial of sepsis (246). Although there was no benefit of eritoran in mortality rates from severe sepsis, there were no differences in adverse events between groups and this may pave the way for further studies of TLR4 modulation for other conditions.

#### TLR5

The role of TLR5 in intestinal inflammation is less clear. TLR5^−/−^ mice develop a spontaneous colitis (247) and mice treated with purified *Salmonella*-derived flagellin as a TLR5 agonist are protected from *Clostridium difficile* colitis (248). Furthermore, the TLR5 agonist CBLB502 has antioxidant actions and scavenges oxygen free radicals (249) through which it may exert anti-inflammatory properties. However, flagellin enemas have been shown to exacerbate established DSS colitis (250) although in a non-TLR5-dependent manner (251), highlighting the need for further studies.

#### TLR7–9

The TLR7 agonist imiquimod, administered orally, induces type-1 IFN responses in the colonic mucosa to ameliorate DSS colitis and has effects *in vitro* on antimicrobial peptide production (252). A further study has confirmed a similar benefit in the TNBS model of colitis extending the mechanism of action to regulation of regulatory T cells (253). The development of 2′-*O*-methyl modifications to RNA has been used to develop TLR7 antagonists (254, 255), which may improve understanding of the signaling pathway in intestinal inflammation.

TLR7, along with TLR8 and 9, is an endosomal PRR (256). Inhibition of both TLR7 and 9 with IMO-3100, an oligonucleotide antagonist, or of TLR7, 8 and 9 with IMO-8400, resulted in a decrease in *IL17A* expression in an IL-23-dependent murine model of skin inflammation (257). Similar effects have been seen in human peripheral blood mononuclear cells (258) and, specifically for IMO-8400, on the induction of psoriatic lesions by inhibiting Th1/17 cytokines (259). Short-term treatment of patients with psoriasis in a phase 2a clinical trial yielded clinical benefit without significant adverse events (260). It is plausible therefore that this approach may yield benefit in Crohn’s disease which is also associated with IL-12/23 dysfunction (261).

TLR9 detects bacterial immunostimulatory DNA sequences. Synthetic DNA oligonucleotides (ODNs) ameliorate colitis whether induced by DSS, hapten, or in the IL10^−/−^ mouse (262). However, it appears that the effects of ODNs are dependent on time of administration relative to induction of colitis. ODNs given therapeutically after colitis has developed worsen disease whereas when given prophylactically there is a reduction in inflammation thought possible due to a tolerance effects on IFNγ or on increasing IL-10 production (263). The same group went on to show that intestinal inflammation following DSS challenge is reduced in TLR9^−/−^ mice compared with wild-type and that administration of adenoviral ODNs (which blocks the effects of DNA sequences) to wild-type mice with established colitis results in amelioration of disease (264). The findings were replicated in the severe combined immunodeficiency disease transfer model of colitis and in the IL10^−/−^ mouse and the authors conclude that DNA sequences from gut microbes perpetuate chronic inflammation through TLR9 and that adenoviral ODNs may be of benefit in intestinal inflammation (264). *In vitro* studies of the c41 ODN, from *Pseudomonas aeruginosa*, in a murine macrophage cell line and in human monocytes demonstrates that this sequence binds to TLR9 without triggering downstream cascades but prevents other ODNs from binding and so has uses as a TLR9 antagonist (265). A subsequent study demonstrated more diverse actions of c41 on TLR activation suggesting wider effects than simply TLR9 (266). Similar to c41, the antimalarial drug chloroquine has diverse effects on TLR signaling but particularly *via* TLR9 to alleviate murine colitis (267).

Unlike many of the TLRs, TLR9 modulation has been studied clinically in patients with ulcerative colitis using a TLR9 agonist, DIMS0150 (also known as cobitolimod/Kappaproct). A single dose instilled to the mucosa of the transverse or descending colon at colonoscopy resulted in 43% clinical response at week 1, 71% at week 4, and 86% at week 12 (268). Remission rates were slightly lower than response but similarly increased to week 12. Clinical scores were mirrored by endoscopic improvement. Note was made of improved steroid sensitivity in patients treated with DIMS0150. An analysis of steroid-response genes identified from the pilot study (*CD163, TSP-1, IL-1RII*) were validated in a prospective cohort of patients treated with rectal placebo or DIMS0150 demonstrating utility of this gene panel in identifying patients most likely to benefit from TLR9 agonist therapy (269). Unfortunately, a larger randomized, double-blind, placebo-controlled trial of 131 patients with ulcerative colitis failed to reach its primary endpoint to demonstrate benefit of DIMS0150 over placebo at week 12 although some benefits were seen at earlier timepoints including on mucosal healing at week 4 (270). The treatment was not associated with serious adverse events and, as the authors conclude, further trials are merited. A second trial is currently in the recruitment stages (NCT03178669).

Similar to DIMS0150, a synthetic oligonucleotide acting as a TLR9 agonist, BL-7040 (previously known as Monarsen and EN101), which can be administered orally may also have potential in the management of human IBD. Originally designed as a therapy for myasthenia gravis due to its effects on acetylcholinesterase transcripts (271), BL-7040 was shown to be an activator of TLR9 signaling to increase levels of indoleamine and IFNα (272). It has subsequently been demonstrated that this compound induces miRNA changes to activate the alternative pathway of NF-κB activation through TLR9 (273). These preclinical findings have been extended to a phase 2a trial in ulcerative colitis, which confirmed the safety of BL-7040 in intestinal inflammation and demonstrated an improvement in colitis in half of the patients who completed the full study protocol (274).

### Other Targets Influencing Innate Immune Signaling in Intestinal Inflammation

Modulation of signaling cascades downstream of TLRs themselves have also shown promise for intestinal inflammation. The MyD88 inhibitor TJ-M2010-5 improved azoxymethane/DSS colitis and consequent colitis-associated colonic cancer (275). Nur77 is a transcription factor which is associated, from GWAS, with IBD and interacts with TRAF6 to interfere with TLR–IL1-R signaling to inhibit NF-κB cytokine production (276). Cytosporone B is an agonist of Nur77 and ameliorates DSS colitis (276).

Apilimod inhibits the lipid kinase activity of phosphatidylinositol-3-phosphate-5-kinase (PIKfyve) resulting in inhibition of IL-12/23p40 (277). Although initially promising, a phase 2 trial of apilimod in 220 patients with active Crohn’s disease failed to show benefit over placebo, although was well tolerated (278). A novel PIKfyve inhibitor, APY0201, administered orally ameliorated intestinal inflammation in the IL10^−/−^ cell transfer model and showed effects *in vitro* on IL-12/23 production in macrophages (279). A more in depth understanding of the PIKfyve pathway may yet yield opportunities for reducing intestinal inflammation.

The narrow spectrum kinase inhibitor TOP1288 targets p38, Src, Lck, and Syk and has been shown to have anti-inflammatory effects relevant for colitis both *in vitro* and *in vivo* (280, 281). Recently, a clinical trial of six patients with active ulcerative colitis despite oral mesalazine was conducted with TOP1288 (282). The drug had minimal systemic bioavailability, was well tolerated and demonstrated promising reductions in colonic IL-6 and 8. Other therapies involving modulation of innate immune cell function under evaluation for intestinal inflammation include laquinimod which exerts at least some effects through effects on antigen presentation (283, 284). A phase 2 clinical trial of laquinimod in Crohn’s disease was safe and demonstrated benefit on clinical remission especially at lower doses (285).

## Conclusion

**Figure 2:**
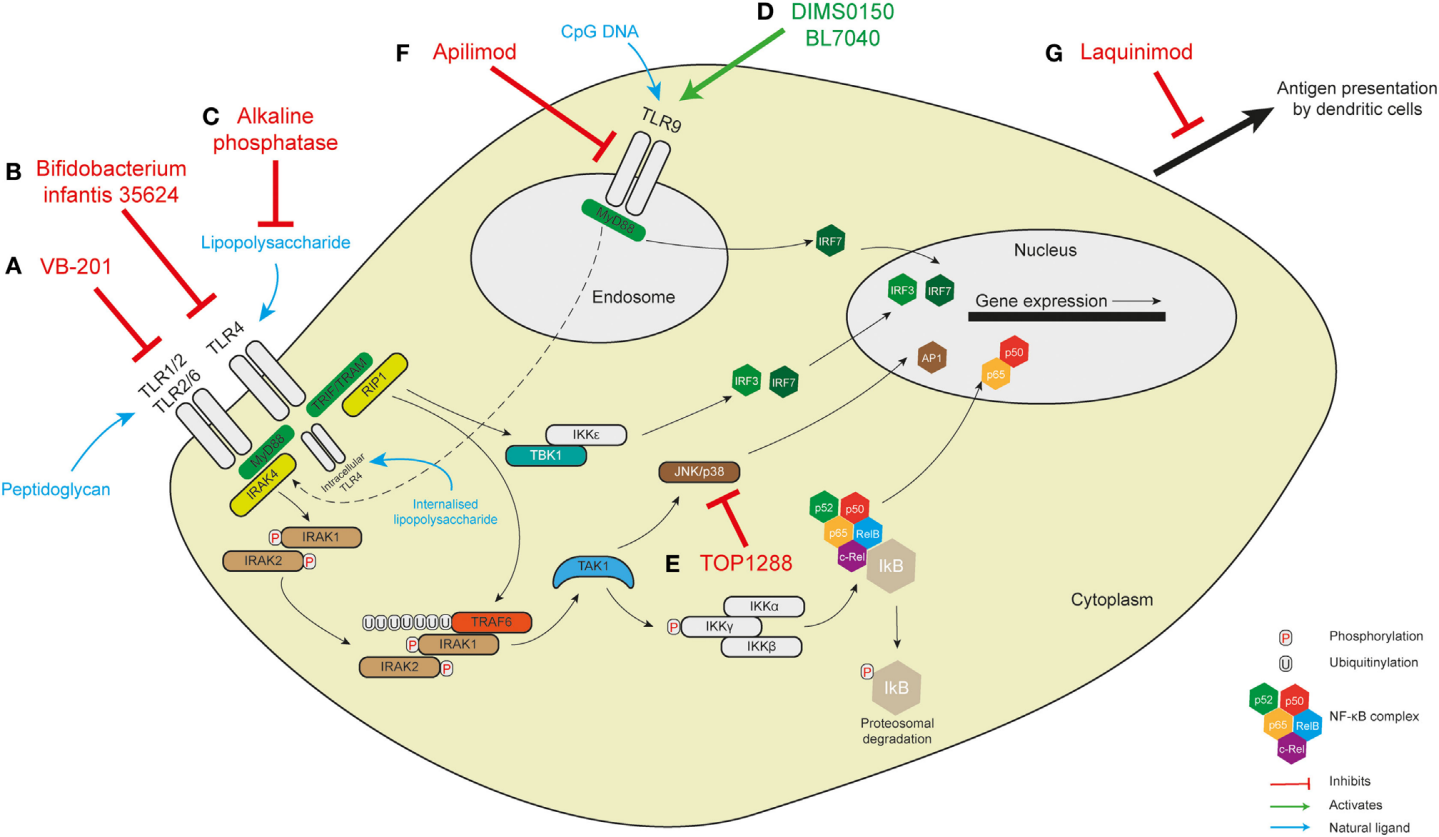
Targeting pattern-recognition receptor signaling in human intestinal inflammation. **(A)** The small molecule VB-201 interferes with downstream signaling from TLR2 and has been trialed in ulcerative colitis. **(B)** Similarly, *Bifidobacterium infantis* 35624, which probably acts to interfere with the TLR4 signaling pathway, has been trialed in maintaining remission in ulcerative colitis. The results of these two trials are not known. TLR4 is found on the cell membrane in immune cells but in intestinal epithelial cells has been demonstrated intracellularly where it responds to internalized lipopolysaccharide. **(C)** Alkaline phosphatase detoxifies LPS to inhibit TLR4 signaling and has been shown to be of benefit in ulcerative colitis. It is not known whether it might interfere with intracellular TLR4. Similar benefits have been seen with the TLR9 agonists DIMS0150 and BL7040 **(D)**. **(E)** TOP1288, a narrow spectrum kinase inhibitor, has effects on p38, Src, Lck, and Syk to reduce colonic IL-6 and -8 in ulcerative colitis. **(F)** The exact role of apilimod in innate immune sensing is not well understood but there are effects on endosomal maturation and TLR9 sensing—this was not, however, effective in a trial in Crohn’s disease. **(G)** Laquinimod exerts some effects on antigen presentation by dendritic cells with some improvement in remission in Crohn’s disease.

Pattern-recognition receptor-mediated control of innate immunity has a fundamental role in both mounting immune defense and maintaining intestinal homeostasis. In this review, we have summarized emerging key functions of this receptor class that, if dysregulated due to functional or genetic defects, may be responsible for the induction of intestinal inflammation such as IBD. Attempts are being made to manipulate PRR-directed pathways for therapeutic purposes (summarized in Table 1 and Figure 2). Although the number of clinical trials that have been performed using this approach is currently small there are a number of compounds in the preclinical evaluation stage. The results from trials performed to date have not been greatly successful in IBD. It is likely a better molecular understanding of PRR biology within human intestinal cells and *in vivo* models is required to harness the full potential of this approach clinically.

**Table 1:** A summary of compounds targeting Toll-like receptors to modulate animal and/or human intestinal inflammation.

Target	Compound	Effect on target	Animal models	Effect on inflammation	Human trials	Effect	Key references
TLR2	Polysaccharide A	Sensed by TLR2	*Helicobacter hepaticus*, 2,4,6-trinitrobenzenesulphonic acid (TNBS), dextran sulfate sodium (DSS)	Improvement			(193–197)
	Purified curli fibers	Sensed by TLR2	TNBS	Improvement			(198)
	Lipoarabinomannan/lipoteichoic acid	Agonist	NSAID-induced ileitis	Improvement			(199)
	TLR2-p	Blockade of dimerization	DSS	Improvement			(201)
	VB-201	Inhibitor			UC (phase 2)	Unknown	Unpublished
TLR3	Poly(I:C)	Agonist	DSS, TNBS	Improvement			(210, 211, 213, 214)
TLR4	Alpinetin	Inhibitor	DSS	Improvement			(188)
	1A6	Inhibitor	DSS	Improvement			(227)
	Non-absorbable polypropyletherimine dendrimer glucosamine	Inhibitor	*Shigella* colitis	Improvement			(229)
	Alkaline phosphatase	Detoxifies LPS	DSS	Improvement	UC (phase 2)	Improvement	(238, 239)
	*Bifidobacterium infantis* 35624	Interferes with TLR4 signaling			UC (phase 2)	Unknown	Unpublished
	Corticotrophin releasing factor deficiency	Downregulated TLR4	DSS	Worsen			(243)
	Baicalin	Interferes with TLR4 signaling	DSS	Improvement			(244)
	Palmitoylethanolamine	Interferes with TLR4 signaling	DSS	Improvement			(245)
TLR5	*Salmonella*-derived flagellin	Agonist	*Clostridium difficile* colitis	Improvement			(248)
TLR7	Imiquimod	Agonist	DSS, TNBS	Improvement			(252, 253)
TLR9	CpG oligodeoxynucleotides (ODNs)	Agonist	DSS, hapten, IL10^−/−^	Improvement or worsen^a^			(262, 263)
	Adenoviral oligonucleotides	Inhibits CpG ODNs	DSS, SCID *transfer, IL10*^−/−^	Improvement			(264)
	Chloroquine	Suppresses TLR2/9 signaling	DSS	Improvement			(267)
	DIMS0150 (Cobitolimod, Kappaproct)	Agonist			UC (phase 3)	Improvement	(268, 270)
	BL7040 (Monarsen, EN101)	Agonist			UC (phase 2)	Improvement	(274)

*^a^Effect dependent on whether prophylactic or therapeutic administration*.

## Author Contributions

All authors contributed equally to the conception and overview of the manuscript content. DC, TC, and TA contributed equally to the writing of the manuscript with AS providing intellectual content and oversight. All authors edited and approved the final version of the manuscript.

## Conflict of Interest Statement

The authors declare that the research was conducted in the absence of any commercial or financial relationships that could be construed as a potential conflict of interest.

## Footnotes

^1^https://www.bsg.org.uk/resource/iaso-trial-of-anakinra-for-acute-severe-ulcerative-colitis.html [accessed 9th Feb 2018].

## Abbreviations

DAMP, damage-associated molecular pattern; DCs, dendritic cells; DSS, dextran sulfate sodium; IBD, inflammatory bowel disease; IEC, intestinal epithelial cell; mtDNA, mitochondrial DNA; MDP, muramyl dipeptide; mROS, mitochondrial reactive oxygen species; NLR, NOD-like receptor; PRR, pattern-recognition receptor; TLR, toll-like receptor; TNBS, 2,4,6-trinitrobenzenesulphonic acid.
